# Detection of mitochondrial insertions in the nucleus (NuMts) of Pleistocene and modern muskoxen

**DOI:** 10.1186/1471-2148-7-67

**Published:** 2007-04-27

**Authors:** Sergios-Orestis Kolokotronis, Ross DE MacPhee, Alex D Greenwood

**Affiliations:** 1 Sackler Institute for Comparative Genomics, American Museum of Natural History, Central Park West at 79^th ^Street, New York, NY 10024, USA; 2 Department of Ecology, Evolution, and Environmental Biology, Columbia University, 1200 Amsterdam Avenue, MC5557, New York, NY 10027, USA; 3 Department of Mammalogy, Division of Vertebrate Zoology, American Museum of Natural History, Central Park West at 79^th ^Street, New York, NY 10024, USA; 4 Department of Biological Sciences, Old Dominion University, Mills Godwin Building, Room 108E, Norfolk, VA 23529-0266, USA

## Abstract

**Background:**

Nuclear insertions of mitochondrial sequences (NuMts) have been identified in a wide variety of organisms. Trafficking of genetic material from the mitochondria to the nucleus has occurred frequently during mammalian evolution and can lead to the production of a large pool of sequences with varying degrees of homology to organellar mitochondrial DNA (mtDNA) sequences. This presents both opportunities and challenges for forensics, population genetics, evolutionary genetics, conservation biology and the study of DNA from ancient samples. Here we present a case in which difficulties in ascertaining the organellar mtDNA sequence from modern samples hindered their comparison to ancient DNA sequences.

**Results:**

We obtained mitochondrial hypervariable region (HVR) sequences from six ancient samples of tundra muskox (*Ovibos moschatus*) that were reproducible but distinct from modern muskox sequences reported previously. Using the same PCR primers applied to the ancient specimens and the primers used to generate the modern muskox DNA sequences in a previous study, we failed to definitively identify the organellar sequence from the two modern muskox samples tested. Instead of anticipated sequence homogeneity, we obtained multiple unique sequences from both hair and blood of one modern specimen. Sequencing individual clones of a >1 kb PCR fragment from modern samples did not alleviate the problem as there was not a consistent match across the entire length of the sequences to *Ovibos *when compared to sequences in GenBank.

**Conclusion:**

In specific taxa, due to nuclear insertions some regions of the mitochondrial genome may not be useful for the characterization of modern or ancient DNA.

## Background

NuMts have been characterized in numerous species [1]. Various genome sequencing projects have demonstrated that many copies ranging in length from parts of genes to nearly full copies of the mtDNA genome exist in most mammalian genomes [2,3]. When identified correctly, older NuMts can be used as a constructional source of phylogenetic outgroups [4]. However, they are better known for their negative impact on the interpretation of collected data. For example, a series of mutations originally thought to be correlated with Alzheimer's syndrome were in fact NuMts [5]. A well publicized sequence obtained from a dinosaur fossil was in fact a NuMt from a human contaminant [6]. Using elephant hair, Greenwood and Pääbo [7] demonstrated that in some species and in some tissues, NuMts may be preferentially retrieved over mtDNA by PCR. More recently, it has been shown that in some primates, specific mtDNA loci may be unusable for phylogeny reconstruction because of NuMts [8-10].

Methods that depend on the circularity of the mtDNA genome, including amplification of long fragments or the entire mtDNA genome, could exclude linear integrated NuMts. However, this generally requires a good source of intact DNA and is not commonly practiced. Given that most non-invasive techniques for retrieving sequence from wild animals require using tissues such as hair or dung containing low concentrations of DNA, often of poor quality, high copy sequences such as those present in mtDNA are often the preferred target for analysis. The problem also applies to degraded DNA such as DNA from ancient samples. Although nuclear DNA is expected to be harder to retrieve from ancient samples, NuMts have been detected therein and such studies are not free from the risks presented by NuMts [11]. For all these reasons, NuMts present a serious challenge.

In our experiments, two primer pairs amplifying overlapping PCR fragments (270 bp and 162 bp, respectively) were applied to both ancient muskox extracts and modern muskox DNA. The sequences were largely uniform among the ancient samples muskoxen although in one sample, among several clones sequenced, one unequivocal NuMt sequence was detected. In contrast to the ancient materials, the same primers yielded multiple distinct sequences from modern muskox hair and blood. Thus, unlike proboscideans, hair and blood both yielded large numbers of NuMts.

An additional PCR product was amplified from the modern materials encompassing the entire HVR. This yielded sequences that were more uniform; however, outside of the region being compared to the ancient samples, the sequences did not match *Ovibos *as well as it matched other related bovids. Phylogenetic analysis of the sequences obtained did not provide clear separation of NuMts into a clade separate from that for organellar mtDNA. Thus, for the specific HVR sequence under study, the organellar mtDNA sequence could not be determined with absolute certitude.

## Results

### Ancient DNA Experiments

**Figure 1:**
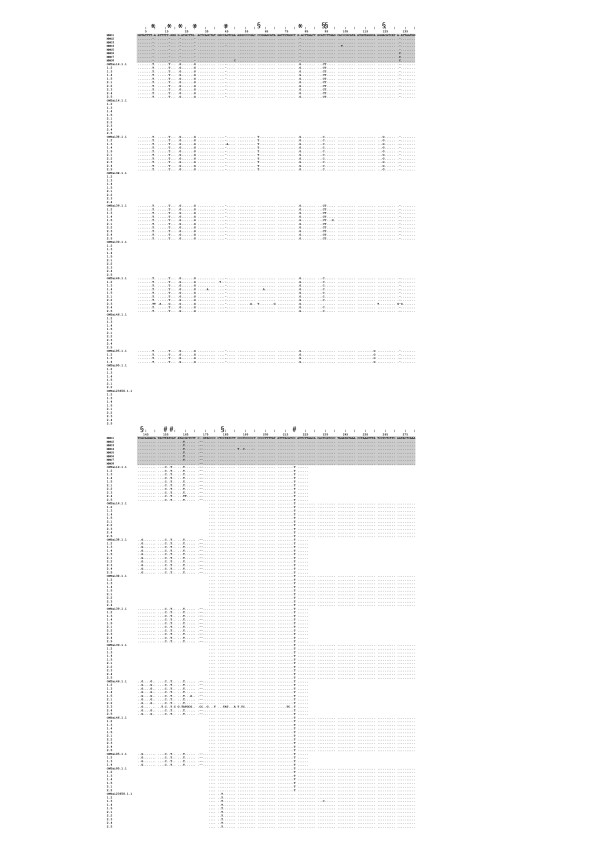
**Clone sequences used to determine ancient muskox mtDNA sequence**. MMO1-8 represent the 8 haplotypes already identified in modern muskoxen [15]. The modern samples are also shaded. Samples are indicated by name (following the naming system in [12]) followed by the PCR repetition number followed by clone number i.e. 1.1 is the first clone sequence from the first PCR amplification. Indels that are in conflict with the published sequences are indicated "*". Base changes that were found only in samples from Taimyr but were not fixed are designated "§". Fixed differences between ancient Taimyr samples, with the exception of OMTai23564, are also indicated "#".

The original intent of our study was to extend the amount of sequence that could be compared between ancient and modern muskoxen beyond what was accomplished by MacPhee *et al*. [12]. Six ancient muskox samples, all deriving from the Taimyr Peninsula (Russian Federation), and ranging in age from 2,970 ± 40 to 44,760 ± 1700 yrbp (radiocarbon years before present), yielded DNA and were amplified with two PCR primer pairs. In one case, OMTai23658, the larger amplification product could not be obtained. This is consistent with the observed poorer quality of the DNA extracted from this sample in previous experiments [12]. For the other specimens, fragments overlapped by 49 bp. Each sample was amplified for each fragment twice and multiple clones per fragment were sequenced (Figure 1). DNA damage is a feature of many ancient DNA samples [13]. However, limited interclone variation was observed. A possible explanation for the lack of interclone variation is that relatively large amplification products could be retrieved reproducibly from other loci such as cytochrome *b *(377 bp) as well, demonstrating the relatively good preservation state of most of the ancient materials used in this study [12].

One clone of sample OMTai46 (clone 2.3 in Figure 1) was highly divergent (differing from the consensus sequence at 32 nucleotide positions). A BLASTN search of this sequence resulted in a highest similarity score to a roe deer control region sequence (*Capreolus capreolus*, e-value = 1e-51) [14]. This finding is typical for a phylogentically older NuMt, such that the sequence divergence from the target taxon's mtDNA sequence places it as more similar, though not identical, to the outgroup. Given the likely age of the NuMt, as suggested by its large divergence from the other clone sequences and its similarity to other caprines, the clone is not a human contamination and likely represents an old NuMt. However, the clone was unique among clones from the same animal, and did not appear in other samples. On the whole, then, NuMt retrieval from the ancient specimens was minimal. This is not surprising as it is expected that, given the high copy number of mtDNA compared to nuclear DNA, in most subfossil samples mtDNA will be greatly in excess over single copy sequence nuclear DNA and therefore preferentially amplified by PCR.

Several indels observed in the ancient samples were not identified in an earlier investigation of modern muskox DNA by Groves [15]. As noted by MacPhee *et al*. [12], these indels are probably artifacts produced by Groves' [15] single-strand direct sequencing protocol. By contrast, when both strands of multiple cloned sequences are investigated, no indels appear (Figure 1). Methodological improvements in sequencing methodology that have taken place since the original publication of the data could explain the discrepancy.

### Modern DNA Experiments

Having observed a NuMt sequence in the ancient DNA, we performed two additional experiments using modern DNA samples. In the first, the same primers used in the investigation of the ancient DNA specimens were applied to samples from two modern muskoxen (both hair and blood from the first, blood only from the second, unrelated individual). In the second experiment, the primers used by Groves [15] to generate her modern muskox database were applied to the ancient samples, which resulted in > 1 kb product (see Additional file 1). Thus, three primer pairs, two tissue types and two individuals were each tested for the presence of NuMts using the same protocol.

All PCR products were cloned and multiple clone sequences determined. The sequences were curated by removing the vector sequence from the reads and the beginning comparisons from the first base following the 3' end of the primers. The most consistent sequences obtained came from the largest fragment investigated (see Additional File 2). Some sequence heterogeneity was observed in the region of overlap for all sequences compared with the exception of one highly divergent clone (CHL.8). Two additional clone sequences, CBL.10 and CHL.5 however, were almost identical to the majority of clones in the region overlapping the shorter PCR products retrieved from ancient DNA but were more divergent in the 5' end of the HVR (see Additional File 2). This extended into the region of the HVR not covered by any of the database muskox sequences. The alignment of the sequences to an outgroup sequence (Taiwanese serow, *Nemorhaedus swinhoei*) and to the database did not generate a consistent alignment across the entire fragment covered by Groves' [15] sequences and those generated in this study. The 3' end of the first fragments from GenBank (U47061, 63, 65, 67, 69, 71, 73 and 75) aligned poorly to the sequences generated in this study and to the outgroup sequence. Similarly, the 5' end of the second fragment sequences from GenBank aligned poorly to the outgroup and to the sequences generated from this study. Thus, the sequences generated in this study aligned consistently to each other, except where noted, and to the outgroup; yet the database muskox sequences did not. Thus, sequence heterogeneity suggesting NuMts were detected among the long fragment sequences and odd behaviour of the database muskoxen was also observed.

Tests for recombination and gene conversion were negative. In addition, whereas in the Groves [15] study 37 muskoxen yielded only 8 distinct haplotypes, the ~1.1 kb fragment analyzed in this study of two individuals yielded two new haplotypes not previously observed. It should be noted that the indels tend to inflate the divergence of the sequences. Most of the differences were base substitutions. Given the presence of insertions and deletions among the ~1.1 kb fragment clones and alignment issues with muskox sequences in the database, we conclude that correct organellar mtDNA sequences cannot be unambiguously determined from the sequencing of the longest PCR product amplified in this study.

**Figure 2:**
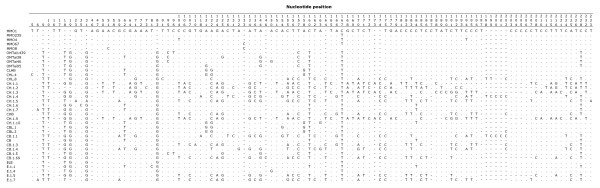
**Polymorphic sites in muskox HVR clone haplotypes**. Each polymorphic nucleotide position is numbered. Nucleotides identical to the first sequence are indicated by a dot and gaps by a dash.

More extreme discrepancies were observed with the PCR products generated with the primers used to amplify from the ancient DNA extracts. The shortest amplified fragment yielded a sequence identical to the database of muskox sequences, and one fixed difference compared to each of the Taimyr muskoxen. Although the sequence obtained for the ~1.1 kb fragment and sequences from the smaller of the two amplification products were very similar to one another, allowing for minor interclone variation, it is not clear whether the smaller fragment is collinear with the ~1.1 kb sequences obtained in this study or one matching the sequence in the database (see Additional File 2). Particularly considering the fact that two of the long fragment clones were almost identical to the database muskoxen and most of the other long clone sequences yet diverged in other portions of the HVR. Also, a relatively recent NuMt sequence might be very similar to sequence in the organellar mtDNA. Conversely, the longer of the two smaller fragments yielded multiple distinct sequences differing from the reference sequence (Figure 2). This result was obtained regardless of the tissue of origin: both hair and blood yielded multiple distinct sequences, with no single type predominating. In addition, none of the sequences in the overlapping region between the two small fragment primer sets matched (data not shown). In contrast, the second muskox blood sample yielded a predominant sequence (6 of 10 clones) that did match in the overlap and matched the sequence obtained with the ~ 1.1 kb fragment for this portion of the HVR. Thus, intraindividual variation in NuMt detection was observed and unlike the case of proboscideans, blood did not yield fewer NuMts than hair [7].

### Evolutionary analyses

**Figure 3:**
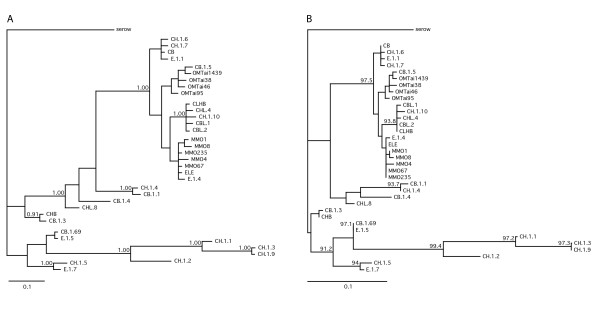
**Phylogenetic reconstructions of muskox HVR haplotypes**. A) Bayesian 50% majority-rule consensus tree. This is a consensus of 18,002 optimal trees after discarding 2,000 trees as burn-in. Numbers above the nodes denote posterior clade probabilities. Values below 0.90 are not shown. Mean Ln*L *= -1239.613. B) Maximum likelihood phylogram. Numbers above the nodes indicate edge support (LRSH) values, e.g. a value of 95 means that the clade is supported at the 0.05 significance level. Only support values above 90 are shown. Ln*L *= -1166.0033. In both trees the bar represents 0.1 substitutions/site along the branches.

In order to determine if the *bona fide *mtDNA sequence for *Ovibos moschatus *could be determined from our material despite the presence of NuMts, various methods of tree reconstruction and different statistical tests were performed. Our main analytical conclusions, with their supporting analyses, are as follows:

1. The chromosomal rearrangement feature enabled in POY is a powerful tool for the establishment of homology between loci differing in their physical arrangement. However, no clone sequence clusters resulted from rearrangement analysis as all combinations had the same cost in a maximum parsimony framework. In addition, no recombination was detectable in this sequence dataset. Thus, the multiple clones derived from different individuals cannot be explained as a PCR artefact or the presence of recombinant mtDNA.

2. The nucleotide frequency distribution across all haplotypes did not reveal any shifts in base composition, so as to hint at a pseudogenization (i.e. the process leading to loss of function of a locus following duplication or transposition), which is mostly expected for coding loci (graph not shown). Up to date, shifts in base composition of D-loop Numts have not been studied, as cases of noncoding Numts have rarely been reported [4,9,10,16-18].

3. Phylogenetic analyses of these results using the alignment in Figure 2 are shown in Figure 3. Sequences CH.1.4 and CB.1.1 were not the most divergent, but they were the only ones sharing a TCCCC insertion at positions 198–202. All phylogenetic analyses -albeit with moderate support- identified two main clades, each with various subclades. The most haplotype-rich clade grouped clones MMO, CHL, CBL, EL, E, OMTai, and a few CB and CH sequences. All three optimality criteria provided strong support (1.00/99.4/100 for BI/ML/MP; see Figure 3 for BI/ML; MP trees not shown) for the clade containing clone sequences CH.1.3, CH.1.9, CH.1.1, and CH.1.2. There was no consensus reached, though, on the phylogenetic position of clades (CB.1.69, E.1.5) and (CHB, CB.1.3). Clade (CB.1.69, E.1.5) was associated with the highly divergent hair clone sequences (CH) with the exception of weighted parsimony that placed it at a basal position within the most haplotype-rich clade. The position of clade (CHB, CB.1.3) was basal but ambiguous, as Bayesian inference and weighted parsimony placed it with the larger clade, while ML placed it with the divergent CH haplotypes, and unweighted parsimony differentiated it as a third clade by itself. We believe this inconsistent clustering is a consequence of the low support at the root of the main clades (53–83% in all methods). Overall, the relationships among haplotypes appear more or less consistent across optimality criteria by maintaining the two main clades with some highly supported subclades.

4. The Pairwise Relative Rates Test identified clones CH.1.3, CH.1.9, CH.1.1, and CH.1.2 to be evolving faster than all other sequences, when compared to all other clones, using the serow as outgroup (see Additional file 3). The RRT also returned significant results for other sequences of the divergent clade, but also for haplotypes of the greater clade. For instance, CH.1.4 evolved at a different rate than same-tissue CH.1.6, CB, or CHB -to name a few- and CB.1.1's rate differed from that of other blood CB and E clones.

5. The unusual haplotype structure and phylogenetic divergence of some CH hair clones, as well as their different evolutionary rate, suggest these sequences are control region fragments transposed to the nucleus. That said, there are other haplotypes that exhibit signs of differential evolution, although they are placed in the main clade and their base composition seems standard, such as CH.1.4, CB.1.1, CHB, CB.1.3. Among the sequences from the long fragment, not all of the clone sequences grouped together. For example, CHL.8 was in a different more basal portion of the tree than any other CHL clone.

In sum, our results are similar to those obtained by Thalmann *et al*. [9], who noted that, in gorillas, NuMts were distributed throughout the phylogenetic tree of HVR sequences. These authors concluded that non-invasive studies relying on the portion of HVR1 examined would not yield reliable results.

Although we were able to identify a set of clones as more divergent even than the outgroup *Nemorhaedus swinhoei*, we were not able to cluster together all clones with unusual sequences, a proof that NuMts may be distributed throughout the phylogenetic tree, rendering phylogenetic detection not the most powerful diagnostic tool.

## Discussion

NuMts are not an insurmountable problem if one is dealing with high quality DNA. Several methods have been suggested for determining correct sequences when confronted by NuMts:

• For mtDNA coding sequences, it is possible to determine the organellar sequence by determining the transcribed mtDNA sequence or for rDNA the proper secondary structure [19,20].

• Overlapping PCR strategies using multiple independent primers operates on the principle that the chances of detecting the same NuMts with independent PCR primers is low. However, this method is not infallible [9].

• Phylogenetic and substitution rate analyses can be used to identify NuMts. However, this may only detect highly divergent NuMts and, as seen in this study, the distribution of sequences did not clearly separate a NuMt clade from an organellar mtDNA clade (Figure 3).

• Amplifying large fragments of mtDNA that overlap from high quality genomic DNA or samples enriched for mtDNA are a good way of detecting organellar DNA. Most NuMts are short, containing one or a few genes. This technique, however, requires a source of high quality DNA or the possibility of enriching samples for mtDNA.

By contrast, studies of ancient DNA or non-invasive sampling often require the use of DNA of substandard quality. In addition, the hypervariable region is often particularly critical for determining variation among individuals that are closely related (i.e., intrapopulation variation). The results of this study demonstrate that even when high quality DNA from fresh blood extractions is available, difficulties in establishing the correct organellar mtDNA sequence may be encountered. In fact, in our example the modern samples proved to be more intractable than the ancient DNA samples.

In the muskoxen samples reviewed here, the frequency of NuMt detection varied among the individuals tested. However, unlike the case with proboscideans [7], both hair and blood from the same individual yielded abundant NuMt sequences. NuMts were also detected, though at lower frequency, in the ancient DNA samples. This makes sense: since only the highest-copy DNA generally remains in significant amounts, the incidence of NuMts should be reduced in ancient samples. Indeed, this inference remains the basic justification for the continued use of mtDNA in most ancient DNA studies. However, because NuMts have also been detected in nuclear DNA derived from ancient samples, there is no avoiding the conclusion that NuMts are a potential problem for all sample types, irrespective of their origin or condition [21].

Direct sequencing of the PCR products would not alleviate the problem: if a NuMt predominates, then the NuMt sequence will be retrieved. Furthermore, the presence of multiple distinct sequences would make direct sequencing impossible. In our example, the largest fragment sequenced demonstrated inconsistent homology with the 8 muskox haplotypes deposited in GenBank [15], despite the fact that the same primers were used throughout. Because of this inconsistency, even though one of the blood samples yielded matching sequences in all overlapping PCR fragments, it cannot be definitively stated that the sequence represented is actually organellar mtDNA. Nor did phylogenetic analysis distinguish NuMts from organellar mtDNA sequences.

## Conclusion

Mitochondrial sequences have proven to be useful in a wide variety of contexts. However, there are some often overlooked risks associated with their use. We have observed significant differences in the retrieval of NuMts from modern and ancient DNA samples of the muskox *Ovibos moschatus*. These differences, which occurred among independent PCRs using different primer pairs, among individuals and among tissues of individuals, challenge our ability to correctly characterize organellar mtDNA sequences in this taxon. *Ovibos *joins proboscideans, some primates, and several other major taxa in which the high incidence of NuMts complicates proper sequence identification. Caution should be exercised in interpreting the results of any mtDNA study that relies on limited or degraded DNA samples or single PCR primer pairs. In extreme cases, the pervasive presence of NuMts may render some loci useless for the purpose of modern and ancient DNA studies.

## Methods

### Samples and DNA extraction

Ancient muskox samples OMTai 14, 38, 39, 46, 95, and 23564, localities, carbon dates and museum identification numbers are described in detail in our previous study (see Table 1 in [12]). Hair and blood from a modern muskox male was sampled from Tierpark Hellabrunn (Munich, Germany). Blood from a second muskox was kindly provided by E. Willerslev (University of Copenhagen, Denmark). Ancient samples were extracted as described in [22]. Modern blood and hair extractions were performed using QIAamp Mini DNA kits (QIAGEN, Germany) and eluted in 500 μl of distilled water.

### PCR, cloning, and sequencing

PCR primers used in this study were designed to amplify the mitochondrial HVR of muskoxen based on the sequences for *Ovibos *in GenBank [15]. To amplify the entire HVR from modern muskoxen, the primers from [15] were used. For both ancient and modern DNA two overlapping PCR fragments were used to obtain an approximately 276 bp fragment of the HVR. Primer sequences were HV.1L 5'-AAAGAATTCTGCTGTCATACATTT-3', HV.1H 5'-AAAGGATCCAGGGATGAGTGTGTT-3', HV.2L 5'-AAAGAATTCTATCATATATGCTCTTCGTA-3', HV.2H 5'-AAAGGATCCTATCTTGGTTGGAGTGCAGA-3'. Ancient DNA PCR buffer and reaction conditions are described in [22]. Modern PCR reactions were performed using 0.8 pmol of each primer and *Taq *DNA polymerase (Promega). The re-amplification procedure, cloning of PCR products and sequencing of clones was done using standard methods and has already been described [22].

### Alignment and sequence analysis

Sequences were aligned in MUSCLE 3.6 [23] and manually adjusted in Se-Al 2.0a11 [23] before being collapsed into haplotypes in COLLAPSE 1.2 [24] treating gaps as 5^th ^state (see Additional file 4). Previously published control region sequences [15] were also included in the alignment (GenBank:U47061–U47076). The haplotype alignment was used in evolutionary analyses. Sequences were submitted to GenBank (GenBank:EF057069–EF057098). The Taiwanese serow *Nemorhaedus swinhoei *(GenBank:AY149639) was used as outgroup, as a closely related caprine [25].

We attempted to cluster homologous clone sequences, so as to differentiate true mitochondrial sequences from Numts, by applying two different classification schemes: (i) clones were categorized by each muskox individual regardless of the number of clones per individual, resembling to a multiple-partitions dataset; the inequality of the number of clones per individual was corrected by coding missing clones as partitions with missing data. (ii) clones were categorized by individual and by tissue type, e.g. hair (H) and blood (B), knowing from previous research [7] that hair may be more Numt-rich than blood. The homology assessment analysis was done in POY [26] using the -chromosome -n2reorder command.

In recent years, evidence has burgeoned for the occurrence of mitochondrial recombination in a wide variety of animal taxa [27,28], and more specifically in members of the family Bovidae, such as *Cephalophus *spp. (duikers), *Ovis *spp. (sheep), *Tragelaphus *spp., and *Kobus *spp (see Table 1 in [28]). Recombination should not produce misleading interpretations of populations with limited or nonexistent gene flow; however, it can mimic the traces of population size expansion, underestimate divergence times, and mask ancient polymorphisms as recurring mutations [27,29]. In the particular case of caprines, in which mtDNA recombination is an acknowledged issue [28], it should be routine to search for evidence of recombination before making evolutionary inferences.

In order to examine the possibility of detecting HVR recombinant sequences, we employed several recombination detection methods, such as Bootscan, Chimaera, Geneconv, MaxChi, RDP, and SiScan, as implemented in the program RDP2 2b08 [30], using the automated scan option, and 1,000 permutations and 1,000 bootstrap replicates where applicable.

We tried to detect changes in base composition across sequences by plotting the relative compositions of individual bases, purines, and pyrimidines across all haplotypes, using a sliding window of 50 bp and a step of 10 bp in Treefinder [31].

The substitution model suggested by MrModeltest 2.2 [32] in conjunction with PAUP* 4b10 [33] using Akaike's Information Criterion (AIC) [34] was the general time-reversible model [35-37] with substitution rates following a Γ-distribution (GTR+Γ_4_) with shape parameter α = 0.3732.

Bayesian inference (BI) of phylogeny was performed for all haplotypes of the short alignment in MrBayes 3.1.2 [38, 39] on a 9-node dual G5 processor (2.0 GHz, 18 GB RAM) XServe cluster at AMNH, using the model suggested by MrModeltest (GTR+Γ, with a flat prior on base frequencies following a Dirichlet distribution and six rate categories). Two simultaneous analyses were run for 10^7 ^generations each, on two separate occasions, starting from different random trees, and the resulting trees were saved every 1000 generations. One "cold" and three "heated" chains were run with heating scheme fine-tuning; temperature parameters ranging from 2 to 0.03 were used and the latter was chosen, as it was leading to better mixing. The heat applied to all chains except for the "cold" one, was 0.97, 0.94, and 0.92, respectively. Successful chain swaps were in the range of 33–75%. Following visual inspection in Tracer 1.3 [40], stationarity of the Ln-likelihood (i.e. the Ln-probability of the data given the parameter values) was reached before 10^6 ^generations. Similarly, we examined the average standard deviation of split frequencies and confirmed it approached zero (0.003418), indicating a satisfactory run length. Subsequently, the first 1,000 trees were discarded as burn-in and a 50% majority-rule consensus tree [41] was built with 18,002 optimal trees. The proportion of resulting trees presenting a given clade, in other words, the posterior probability of this clade, represents clade support in a Bayesian phylogenetic analysis [42].

Phylogeny was also estimated in a maximum likelihood (ML) framework in Treefinder using the GTR+Γ_4 _model. Approximate bootstrap support was estimated by applying the Shimodaira-Hasegawa (SH) test with RELL approximation [43] to all local rearrangements around an edge on the topology (also referred to as edge support or LRSH) and 50,000 replicates. For every edge on the tree, all its adjacent branch nearest-neighbor interchanges are computed and edge lengths are re-estimated by fixing all other parameters, and finally the SH test is applied. LRSH support values are the complement of the worst *p*-values from the SH test. A support value of 99 means this clade is significant at the 0.01 level, a value of 95 at the 0.05 level, and so on.

We employed unweighted and weighted maximum parsimony (MP) in PAUP* with a heuristic search using 100 random addition sequence replicates and TBR branch swapping. Given the number of transitions and transversions as calculated in MacClade 4.08 [44] (88 transitions and 22 transversions) yielding a transition-transversion ratio of 4:1, we downweighted transitions four times using a step-matrix. In unweighted parsimony, gaps were treated as missing data and as a 5^th ^state. Clade support was provided with 500 bootstrap replicates [45] and strict consensus trees [46] were built using all equally most parsimonious trees.

We used a Pairwise Relative Rates Test [47] to detect sequences with significantly different evolutionary rates, as implemented in HyPhy 0.99b [48] with a GTR+Γ substitution model and a "Local" model. Given a predefined outgroup sequence, the maximum likelihood estimate (MLE) for each 3-taxon tree is calculated, followed by the MLE calculation for the 3-taxon trees with evolutionary rates along the ingroup sequences constrained to be equal. A likelihood-ratio test (LRT) is then performed to examine if rates are equal or independent. The desired precision (absolute error) in the calculation of the Ln-likelihood value was set at 0.001.

## Authors' contributions

ADG was responsible for the experimental work and participated in the sequence data analysis. SOK performed the sequence and evolutionary analyses. RDEM obtained and arranged for the dating of all ancient muskox specimens. All authors co-wrote the manuscript, read, and approved the final draft.

## Supplementary Material

Additional file 1 **PCR products generated from hair and blood of modern muskoxen**. An example of the three PCR products amplified from hair and blood of two muskoxen. An ethidium bromide stained 2% agarose gel is shown with a ladder for each reaction. PCR A, B, and C correspond to primer combinations HV.1L+HV.1H, HV.2L+HV.2H, and published muskox primers [15], respectively. 1, water negative control, 2, ladder, 3, male muskox hair DNA, 4, same muskox blood DNA, 5, a second unrelated muskox blood DNA sample. Click here for file

Additional file 2 **Alignments of the overlapping regions of the 1.1 kb PCR products to muskox control region sequences from GenBank**. Long PCR fragments are aligned to short PCR fragments from modern and Pleistocene muskoxen, as well as all *Ovibos moschatus *control region sequences retrieved from GenBank and the serow control region sequence from GenBank. Names describe muskox individual (C or E), tissue of origin (H for hair, B for blood), and clone number. For additional sequences included, see Additional file 4 for haplotype descriptions. Clones CBL.10 and CHL.5 aligned very poorly at the 5'-end of the sequence and were trimmed. The sequences extend beyond what is shown. Nucleotides identical to the first sequence are indicated by a dot and gaps/missing data by a dash. Long fragment sequences were submitted to GenBank (GenBank: EF566449–EF566462). Click here for file

Additional file 3 **Pairwise Relative Rate Test results**. Significant likelihood ratio tests (LRT) of pairwise combinations of sequences evolving at different rates. The evolutionary rate of the outgroup was independently calculated in all 3-taxon trees. Branch lengths denote the number of substitutions per site. Results are listed by ascending order of significance. Click here for file

Additional file 4 **Explanation of haplotypes**. Collapsed sequences into haplotypes treating gaps as 5^th ^state. The frequency is calculated within the same individual muskox and clone type (e.g. hair or blood). Click here for file
