# Supplementary figures and images for: Detection of mitochondrial insertions in the nucleus (NuMts) of Pleistocene and modern muskoxen
Source: BMC Evol Biol. 2007 Apr 27;7:67. doi: 10.1186/1471-2148-7-67 (PMC1876215; doi:10.1186/1471-2148-7-67)

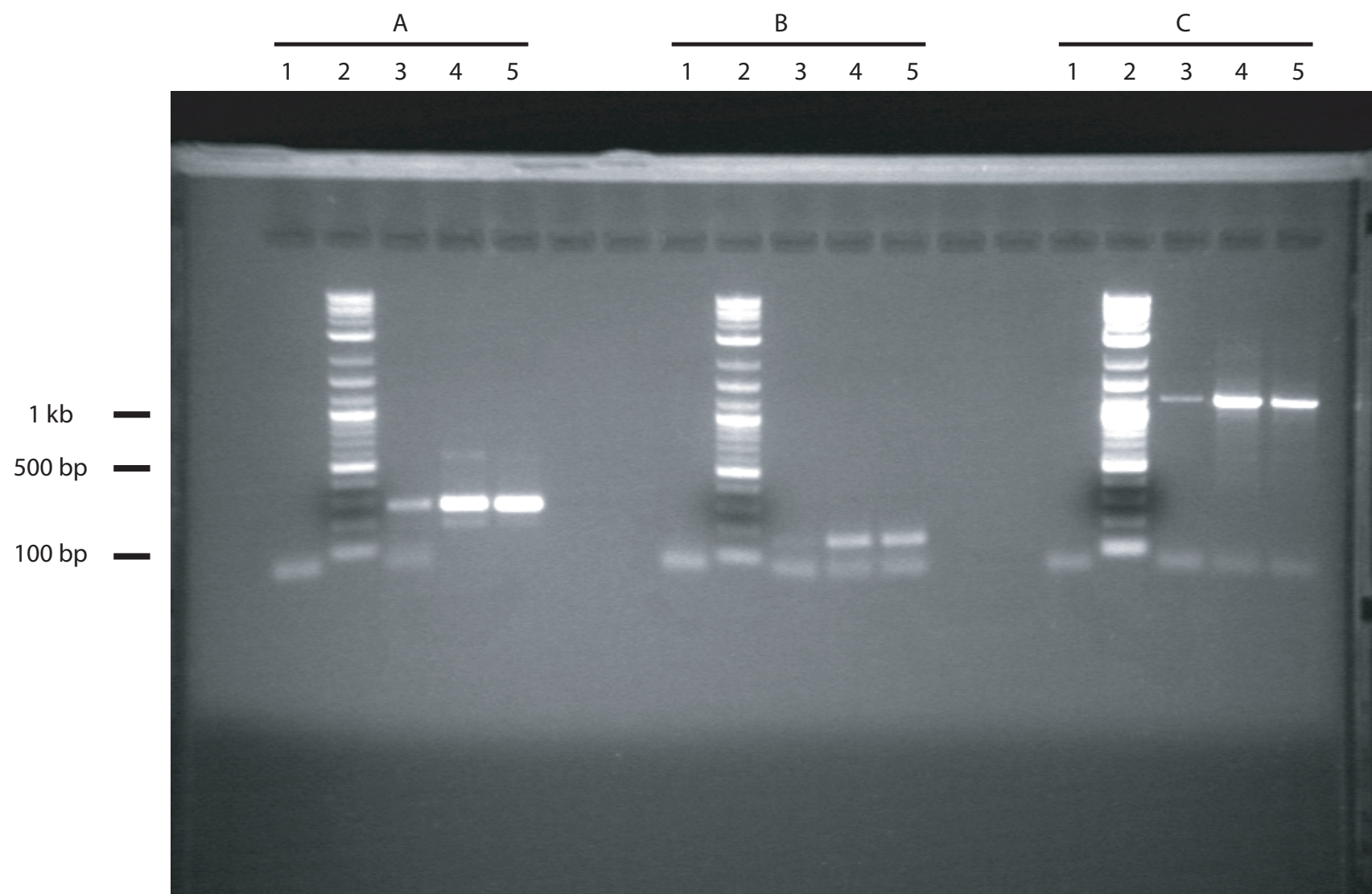

Supplement: Additional file 1 — PCR products generated from hair and blood of modern muskoxen. An example of the three PCR products amplified from hair and blood of two muskoxen. An ethidium bromide stained 2% agarose gel is shown with a ladder for each reaction. PCR A, B, and C correspond to primer combinations HV.1L+HV.1H, HV.2L+HV.2H, and published muskox primers [15], respectively. 1, water negative control, 2, ladder, 3, male muskox hair DNA, 4, same muskox blood DNA, 5, a second unrelated muskox blood DNA sample. [file 1471-2148-7-67-S1.pdf]
