# Supplementary material for: Detection of mitochondrial insertions in the nucleus (NuMts) of Pleistocene and modern muskoxen
Source: BMC Evol Biol. 2007 Apr 27;7:67. doi: 10.1186/1471-2148-7-67 (PMC1876215; doi:10.1186/1471-2148-7-67)
Supplement: Additional file 2 — Alignments of the overlapping regions of the 1.1 kb PCR products to muskox control region sequences from GenBank. Long PCR fragments are aligned to short PCR fragments from modern and Pleistocene muskoxen, as well as all Ovibos moschatus control region sequences retrieved from GenBank and the serow control region sequence from GenBank. Names describe muskox individual (C or E), tissue of origin (H for hair, B for blood), and clone number. For additional sequences included, see Additional file 4 for haplotype descriptions. Clones CBL.10 and CHL.5 aligned very poorly at the 5'-end of the sequence and were trimmed. The sequences extend beyond what is shown. Nucleotides identical to the first sequence are indicated by a dot and gaps/missing data by a dash. Long fragment sequences were submitted to GenBank (GenBank: EF566449–EF566462). [file 1471-2148-7-67-S2.pdf]

```
                              5          15         25         35         45         55         65         75         85         95         105        115        125        135        145        155        165        175        185        195
CBL.6            GAGAACAACC AACCTCCCCA AGACTTCAAG GAAGAAGCTA TAGCTCCACT ATCAACACCC AAAGCTGAAG TTCTATTTAA ACTACTCCCT GAATTACCAC TAACACCTTC TATTAATATA CCACTAAAAA CATTAAGAGC CTTCCCAGTA TCAAATCTAC CAAAACTTTC AATAATATAA CACTAATTTC CTACTCCACA
CBL.7            .......... .......... .......... .......... .......... .......... .......... .......... .......... .......... .......... .......... .......... .......... .......... .......... .......... .......... .......... ..........
CBL.8            .......... .......... .......... .......... .......... .......... .......... .......... .......... .......... .......... .......... .......... .......... .......... .......... .......... .......... .......... ..........
CBL.10           ---------- ---------- ---------- ---------- ---------- ---------- ---------- ---------- ---------- ---------- ---------- ---------- ---------- ---------- ---------- ---------- ---------- ---------- ---------- ----------
CHL.4            .......... .......... .......... .......... .........C .......... .......... .......... .......... .......... .......... .......... .......... .......... .......... .......... .......... .......... .......C.. ..........
CHL.5            ---------- ---------- ---------- ---------- ---------- ---------- ---------- ---------- ---------- ---------- ---------- ---------- ---------- ---------- ---------- ---------- ---------- ---------- ---------- ----------
CHL.6            .......... .......... .......... .......... .......... .......... .......... .......... .......... .......... .......... .......... .......... .......... .......... .......... .......... .......... .......... ..........
CHL.7            .......... .......... .......... .......... .......... .......... .......... .......... .......... .......... .......... .......... .......... .......... .......... .......... .......... .......... .......... ..........
CHL.8            .......... .......... .......... .......... .......... .......... .......... .......... .......... .......... .......... .......... .......... .......... .......... .......... .......... .......... .......... ..........
EL.5             .......... .G........ .......... .......... .......... .......... .......... .......... .......... .......... .......... .......... .......... .......... .......... .......... .......... .......... .......... ..........
EL.6             .......... .G........ .......... .......... .......... .......... .......... .......... .......... .......... .......... .......... .......... .......... .......... .......... .......... .......... .......... ..........
EL.7             .......... .G........ .......... .......... .......... .......... .......... .......... .......... .......... .......... .......... .......... .......... .......... .......... .......... .......... .......... ..........
EL.8             .......... .G........ .......... .......... .......... .......... .......... .......... .......... .......... .......... .......... .......... .......... .......... .......... .......C.. .......... .......... ..........
EL.10            .......... .G........ .......... .......... .......... .......... .......... .......... .......... .......... .......... .......... .......... .......... .......... .......... .......... .......... .......... ..........
U47075           .......... .G........ .......... .......... .......... .......... .......... .......... .......... .......... .......... .......... .......... .......... .......... .......... .......... .......... .......... ..........
U47073           .......... .G........ .......... .......... .......... .......... .......... .......... .......... .......... .......... .......... .......... .......... .......... .......... .......... .......... .......... ..........
U47071           .......... .G........ .......... .......... .......... .......... .......... .......... .......... .......... .......... .......... .......... .......... .......... .......... .......... .......... .......... ..........
U47069           -----..... .G........ .......... .......... .......... .......... .......... .......... .......... .......... .......... .......... .......... .......... .......... .......... .......... .......... .......... ..........
U47067           -......... .G........ .......... .......... .......... .......... .......... .......... .......... .......... .......... .......... .......... .......... .......... .......... .......... .......... .......... ..........
U47065           --------.. .G........ .......... .......... .......... .......... .......... .......... .......... .......... .......... .......... .......... .......... .......... .......... .......... .......... .......... ..........
U47063           --------.. .G........ .......... .......... .......... .......... .......... .......... .......... .......... .......... .......... .......... .......... .......... .......... .......... .......... .......... ..........
U47061           --------.. .G........ .......... .......... .......... .......... .......... .......... .......... .......... .......... .......... .......... .......... .......... .......... .......... .......... .......... ..........
U47076           ---------- ---------- ---------- ---------- ---------- ---------- ---------- ---------- ---------- ---------- ---------- ---------- ---------- ---------- ---------- ---------- ---------- ---------- ---------- ----------
U47074           ---------- ---------- ---------- ---------- ---------- ---------- ---------- ---------- ---------- ---------- ---------- ---------- ---------- ---------- ---------- ---------- ---------- ---------- ---------- ----------
U47072           ---------- ---------- ---------- ---------- ---------- ---------- ---------- ---------- ---------- ---------- ---------- ---------- ---------- ---------- ---------- ---------- ---------- ---------- ---------- ----------
U47070           ---------- ---------- ---------- ---------- ---------- ---------- ---------- ---------- ---------- ---------- ---------- ---------- ---------- ---------- ---------- ---------- ---------- ---------- ---------- ----------
U47068           ---------- ---------- ---------- ---------- ---------- ---------- ---------- ---------- ---------- ---------- ---------- ---------- ---------- ---------- ---------- ---------- ---------- ---------- ---------- ----------
U47066           ---------- ---------- ---------- ---------- ---------- ---------- ---------- ---------- ---------- ---------- ---------- ---------- ---------- ---------- ---------- ---------- ---------- ---------- ---------- ----------
U47064           ---------- ---------- ---------- ---------- ---------- ---------- ---------- ---------- ---------- ---------- ---------- ---------- ---------- ---------- ---------- ---------- ---------- ---------- ---------- ----------
U47062           ---------- ---------- ---------- ---------- ---------- ---------- ---------- ---------- ---------- ---------- ---------- ---------- ---------- ---------- ---------- ---------- ---------- ---------- ---------- ----------
CHLB             ---------- ---------- ---------- ---------- ---------- ---------- ---------- ---------- ---------- ---------- ---------- ---------- ---------- ---------- ---------- ---------- ---------- ---------- ---------- ----------
ELE              ---------- ---------- ---------- ---------- ---------- ---------- ---------- ---------- ---------- ---------- ---------- ---------- ---------- ---------- ---------- ---------- ---------- ---------- ---------- ----------
CH.1.3           ---------- ---------- ---------- ---------- ---------- ---------- ---------- ---------- ---------- ---------- ---------- ---------- ---------- ---------- ---------- ---------- ---------- ---------- ---------- ----------
CB               ---------- ---------- ---------- ---------- ---------- ---------- ---------- ---------- ---------- ---------- ---------- ---------- ---------- ---------- ---------- ---------- ---------- ---------- ---------- ----------
OMTai38          ---------- ---------- ---------- ---------- ---------- ---------- ---------- ---------- ---------- ---------- ---------- ---------- ---------- ---------- ---------- ---------- ---------- ---------- ---------- ----------
OMTai46          ---------- ---------- ---------- ---------- ---------- ---------- ---------- ---------- ---------- ---------- ---------- ---------- ---------- ---------- ---------- ---------- ---------- ---------- ---------- ----------
OMTai95          ---------- ---------- ---------- ---------- ---------- ---------- ---------- ---------- ---------- ---------- ---------- ---------- ---------- ---------- ---------- ---------- ---------- ---------- ---------- ----------
OMTai139         ---------- ---------- ---------- ---------- ---------- ---------- ---------- ---------- ---------- ---------- ---------- ---------- ---------- ---------- ---------- ---------- ---------- ---------- ---------- ----------
serow_AY149639   A.TC..T... ..TA..A.TT .TCAA.AT.T CCCC..AAAT ATTAAGAG.C TC.CCAGTAT T..AT.A.CC AAAAT..CT. ..A.TA.AAC ACTAACTTC. C.CTC.ACA. C.A...C.CG .TCTC.T.G. ..CAGTATAA TG.A.A.TGG .T.C.CA.GT ACGTGG.ACA T.ATT.TA.T .CACCTGAAT AGTACAT.G.

                              205        215        225        235        245        255        265        275        285        295        305        315        325        335        345        355        365        375        385        395
CBL.6            CGCAACAACG CATGATGGAC GTAATATATG TTTAGTACAC AACCCATAAT CTTAACCCAT TAA-ATAGCA CGTAGACATA ATGTAACACA AGCGTGCTAC GTGCGTAGTA CATTAAGTGG CTTTCCCCAT GCG-TATAAG CATGTACATA AATATTAA-T GTAACAAGGA CATGATATGT ATTATAGTAC ATTAAATGAT
CBL.7            .......... .......... .......... .C........ .......... .......... ...-...... .......... .......... .........T .......... .......... .......... ...-.....-. .......... ........-. .......... .......... .......... ..........
CBL.8            .......... .......... .......... .......... .......... .......... ...-...... .......... .......... .........T .......... .......... .......... ...-...... .......... ........-. .......... .......... .......... ..........
CBL.10           ---------- ---------- ---------- ---------- ---------- ---------- ---------- --------.. .......... GA.A.AG..T ..ATA..CC. .....CA..A ...G...... ..A-...... ..A.C..... ..C.....-. ....T.TA.. ...A...... ..ATAGTACA T.AC.CC...
CHL.4            .......... .......... .......... .......... .......... .......... ...-...... .......... .......... .........T .......... .......... .......... ...-...... .......... ........-. .......... .......... .......... ..........
CHL.5            ---------- ---------- ---------- ---------- ---------- ---------- ---------- --------.. .......... GA.A.AA..T ..ATT..... ......A..A T.......-- ---------- ---------- ---------- ---------- ---------- ---------- ----------
CHL.6            .......... .......... .......... .......... .......... .......... ...-...... .......... .......... .........T .......... .......... .......... ...-...... .......... ........-. .......... .......... .......... ..........
CHL.7            .......... .......... .......... .......... .......... .......... ...-...... .......... .......... .........T .......... .......... .......... ...-...... .......... ........-. .A........ .......... .......... ..........
CHL.8            .......... .......... .......... .......... .......... .......... ...-...... .......... .......... .........T .......... .......... .......... ...-...... .......... ........-. .......... .......... .......... ..........
EL.5             .......... .......... .......... ........G. .......... .......... ...-...... .......... .......... ....C....T ........C. ......A... T..C...... ...-...... .......... .G......-. ....T..... .......... .......... ..........
EL.6             .......... .......... .......... ........G. .......... .......... ...-...... .......... .......... ....C....T ........C. ......A... T..C...... ...-...... .......... .G......-. ....T..... .......... .......... ..........
EL.7             .......... .......... .......... ........G. .......... .......... ...-...... .......... .......... ....C....T ........C. ......A... T..C...... ...-...... .......... .G......-. ....T..... .......... .......... ..........
EL.8             .......... .......... .......... ........G. .......... .......... ...-...... .......... .......... ....C....T ........C. ......A... T..C...... ...-...... .......... .G......-. ....T..... .......... .......... ..........
EL.10            .......... .......... .......... ........G. .......... .......... ...-...... .......... .......... ....C....T ........C. ......A... T..C...... ...-...... .......... .G......-. ....T..... .......... .......... ..........
U47075           .......... .......... .......... ........G. .......... .......... ...T...... .........- .......... ....C....T ......-.C. -T.AGTCGA. ...A...... ..-A...C.. ........-- ---------- ---------- ---------- ---------- ----------
U47073           .......... .......... .......... ........G. .......... .......... ...T...... .........- .......... ....C....T ......-.C. -T.AGTCGA. ...A...... ...A...C-- ---------- ---------- ---------- ---------- ---------- ----------
U47071           .......... .......... .......... ........G. .......... .......... ...C...... .........- .......... ....C....T ......-.C. -T.AGTCGA. ...A...... ..-A...C.. .--------- ---------- ---------- ---------- ---------- ----------
U47069           .......... .......... .......... ........G. .......... .......... ...C...... .........- .......... ....C....T ......-.C. -T.AGTCGA. ...A...... ...A...C.. .......--- ---------- ---------- ---------- ---------- ----------
U47067           .......... .......... .......... ........G. .......... .......... ...T...... ..C......- .......... ....C....T ......-.C. -T.AGTCGA. ...A...... ...A.----- ---------- ---------- ---------- ---------- ---------- ----------
U47065           .......... .......... .......... ........G. .......... .......... ...T...... .........- .......... ....C....T ......-.C. -T.AGTCGA. ...A...... ...A...C.. .........T .TG....CTA ..-------- ---------- ---------- ----------
U47063           .......... .......... .......... ........G. .......... .......... ...C...... .........- .......... ....C....T ......-.C. -T.AGTCGA. ...A...... ..-A...C.. .........T .TG....CTA ..-------- ---------- ---------- ----------
U47061           .......... .......... .......... ........G. .......... .......... ...C...... .........- .......... ....C....T ......-.C. -T.AGTCGA. ...A...... ..-A...C.. .........T .TG....CT- ---------- ---------- ---------- ----------
U47076           ---------- ---------- ---------- ---------- ---------- ---------- ---------- ---------- ---------- ---------- ---------- ---------- ---------- ---------- ---------- ---------- ---------- ---------- ---------- ----------
U47074           ---------- ---------- ---------- ---------- ---------- ---------- ---------- ---------- ---------- ---------- ---------- ---------- ---------- ---------- ---------- ---------- ---------- ---------- ---------- ----------
U47072           ---------- ---------- ---------- ---------- ---------- ---------- ---------- ---------- ---------- ---------- ---------- ---------- ---------- ---------- ---------- ---------- ---------- ---------- ---------- ----------
U47070           ---------- ---------- ---------- ---------- ---------- ---------- ---------- ---------- ---------- ---------- ---------- ---------- ---------- ---------- ---------- ---------- ---------- ---------- ---------- ----------
U47068           ---------- ---------- ---------- ---------- ---------- ---------- ---------- ---------- ---------- ---------- ---------- ---------- ---------- ---------- ---------- ---------- ---------- ---------- ---------- ----------
U47066           ---------- ---------- ---------- ---------- ---------- ---------- ---------- ---------- ---------- ---------- ---------- ---------- ---------- ---------- ---------- ---------- ---------- ---------- ---------- ----------
U47064           ---------- ---------- ---------- ---------- ---------- ---------- ---------- ---------- ---------- ---------- ---------- ---------- ---------- ---------- ---------- ---------- ---------- ---------- ---------- ----------
U47062           ---------- ---------- ---------- ---------- ---------- ---------- ---------- ---------- ---------- ---------- ---------- ---------- ---------- ---------- ---------- ---------- ---------- ---------- ---------- ----------
CHLB             ---------- ---------- ---------- ---------- ---------- ---------- ---------- ---------- ---------- ---------- ---------- ---------- ---------- ---------- ---------- ---------- ---------- ---------- ---------- ----------
ELE              ---------- ---------- ---------- ---------- ---------- ---------- ---------- ---------- ---------- ---------- ---------- ---------- ---------- ---------- ---------- ---------- ---------- ---------- ---------- ----------
CH.1.3           ---------- ---------- ---------- ---------- ---------- ---------- ---------- ---------- ---------- ---------- ---------- ---------- ---------- ---------- ---------- ---------- ---------- ---------- ---------- ----------
CB               ---------- ---------- ---------- ---------- ---------- ---------- ---------- ---------- ---------- ---------- ---------- ---------- ---------- ---------- ---------- ---------- ---------- ---------- ---------- ----------
OMTai38          ---------- ---------- ---------- ---------- ---------- ---------- ---------- ---------- ---------- ---------- ---------- ---------- ---------- ---------- ---------- ---------- ---------- ---------- ---------- ----------
OMTai46          ---------- ---------- ---------- ---------- ---------- ---------- ---------- ---------- ---------- ---------- ---------- ---------- ---------- ---------- ---------- ---------- ---------- ---------- ---------- ----------
OMTai95          ---------- ---------- ---------- ---------- ---------- ---------- ---------- ---------- ---------- ---------- ---------- ---------- ---------- ---------- ---------- ---------- ---------- ---------- ---------- ----------
OMTai139         ---------- ---------- ---------- ---------- ---------- ---------- ---------- ---------- ---------- ---------- ---------- ---------- ---------- ---------- ---------- ---------- ---------- ---------- ---------- ----------
serow_AY149639   ..TG.TGTAA ..CA.AC.TA .C.TGTACGT AG..CATT.A .TGGTT.CCC TCATG.GTG. A.GC.GGTAC ATA.ATAC.. ........A. GA.A.AG..T ..ATTA.... ......A..A .......... ..A-...... .......... ........-. ....T..A.. ...A...... ....AGT-.. ..........
```

```
                  405        415        425        435        445        455        465        475        485        495        505        515        525        535        545        555        565        575        585        595
CBL.6             TGTCCTCCCG CATATAAGCA AGTACATTCG AATTCAGTGA TAGTACATAG TACATTCTAA TGTTTACTCG TACATGGCAC ATTCGAGTCA AA-TCAATTC TTGTCAACAT GCGTATCCCT TCCACTAGAT CACGAGCTTA ATTACCATGC CGCGTGAAAC CAGCAACCCG CTTGGCAGGG ATCCCTCTTC TCGCTCCGGG
CBL.7             .......... .......... .......... .......... .......... .......... .......... .......... .......... ..-....... .......... .......... .......... .......... .......... .......... .......... .......... .......... ..........
CBL.8             .......... .......... .......... .......... .......... .......... .......... .......... .......... ..-....... .......... .......... .......... .......... .......... .......... .......... .......... .......... ..........
CBL.10            .TA..C.A.A .......... .......G-A ..CCT.T... .......... G...-.A... CTG..CA... .....A.... ..C.A..... ..CC.C.... .......... ..A......G C...T..... .......... .CG....... .A........ A......... .......A.. ......G... ......T..A
CHL.4             .......... .......... .......... .......... .......... .......... .......... .......... .......... ..-....... .......... .......... .......... .......... .......... .......... .......... .......... .......... ..........
CHL.5             -------TT. .......... .....GG..A C.....A.A. C......... G.....T..G ..C..GT... .....A.T.. ...GA..... ..-..CG.C. .......... .......... ....T..... .......... GCG....... .......... .......... .......... .......... ..........
CHL.6             .......... .......... .......... .......... .......... .......... .......... .......... .......... ..-....... .......... .......... .......... .......... .......... .......... .......... .......... .......... ..........
CHL.7             .......... .......... .......... .......... .......... .......... .......... .......... .......... ..-....... .......... .......... .......... .......... .......... .......... .......... .......... .......... ..........
CHL.8             .......... .......... .......... .......... .......... .......... .......... .......G.. .......... ..-....... .......... .......... .......... .......... .......... .......... .......... .......... .......... ..........
EL.5              .....C.... .G........ ........T. .G......A. .......... ......T... .......... .G........ .......... ..-...GCC. .......... .......... .......... .......... .......... .......... .......... .......... .........T ..........
EL.6              .....C.... .G........ ........T. .G......A. .......... ......T... .......... .G........ .......... ..-...GCC. .......... .......... .......... .......... .......... .......... .......... .......... .......... ..........
EL.7              .....C.... .G........ ........T. .G......A. .......... ......T... .......... .G........ .......... ..-...GCC. .......... .......... .......... .......... .......... .......... .......... .......... .......... ..........
EL.8              .....C.... .G........ ........T. .G......A. .......... ......T... .......... .G........ .......... ..-...GCC. .......... .......... .......... .......... .......... .......... .......... .......... .......... ..........
EL.10             .....C.... .G........ ........T. .G......A. .......... ......T... .......... .G........ .......... ..-...GCC. .......... .......... .......... .......... .......... .......... .......... .......... .......... ..........
U47075            ---------- ---------- ---------- ---------- ---------- ---------- ---------- ---------- ---------- ---------- ---------- ---------- ---------- ---------- ---------- ---------- ---------- ---------- ---------- ----------
U47073            ---------- ---------- ---------- ---------- ---------- ---------- ---------- ---------- ---------- ---------- ---------- ---------- ---------- ---------- ---------- ---------- ---------- ---------- ---------- ----------
U47071            ---------- ---------- ---------- ---------- ---------- ---------- ---------- ---------- ---------- ---------- ---------- ---------- ---------- ---------- ---------- ---------- ---------- ---------- ---------- ----------
U47069            ---------- ---------- ---------- ---------- ---------- ---------- ---------- ---------- ---------- ---------- ---------- ---------- ---------- ---------- ---------- ---------- ---------- ---------- ---------- ----------
U47067            ---------- ---------- ---------- ---------- ---------- ---------- ---------- ---------- ---------- ---------- ---------- ---------- ---------- ---------- ---------- ---------- ---------- ---------- ---------- ----------
U47065            ---------- ---------- ---------- ---------- ---------- ---------- ---------- ---------- ---------- ---------- ---------- ---------- ---------- ---------- ---------- ---------- ---------- ---------- ---------- ----------
U47063            ---------- ---------- ---------- ---------- ---------- ---------- ---------- ---------- ---------- ---------- ---------- ---------- ---------- ---------- ---------- ---------- ---------- ---------- ---------- ----------
U47061            ---------- ---------- ---------- ---------- ---------- ---------- ---------- ---------- ---------- ---------- ---------- ---------- ---------- ---------- ---------- ---------- ---------- ---------- ---------- ----------
U47076            ---------- ---------- ---------- ---------- ---------- ---------- ---------- ---------- ---------- ---------- ---------- ---------- ---------- ---------- ---------- ---------- ---------- ---------- ---------- ----------
U47074            ---------- ---------- ---------- ---------- ---------- ---------- ---------- ---------- ---------- ---------- ---------- ---------- ---------- ---------- ---------- ---------- ---------- ---------- ---------- ----------
U47072            ---------- ---------- ---------- ---------- ---------- ---------- ---------- ---------- ---------- ---------- ---------- ---------- ---------- ---------- ---------- ---------- ---------- ---------- ---------- ----------
U47070            ---------- ---------- ---------- ---------- ---------- ---------- ---------- ---------- ---------- ---------- ---------- ---------- ---------- ---------- ---------- ---------- ---------- ---------- ---------- ----------
U47068            ---------- ---------- ---------- ---------- ---------- ---------- ---------- ---------- ---------- ---------- ---------- ---------- ---------- ---------- ---------- ---------- ---------- ---------- ---------- ----------
U47066            ---------- ---------- ---------- ---------- ---------- ---------- ---------- ---------- ---------- ---------- ---------- ---------- ---------- ---------- ---------- ---------- ---------- ---------- ---------- ----------
U47064            ---------- ---------- ---------- ---------- ---------- ---------- ---------- ---------- ---------- ---------- ---------- ---------- ---------- ---------- ---------- ---------- ---------- ---------- ---------- ----------
U47062            ---------- ---------- ---------- ---------- ---------- ---------- ---------- ---------- ---------- ---------- ---------- ---------- ---------- ---------- ---------- ---------- ---------- ---------- ---------- ----------
CHLB              ---------- ---------- ---------- ---------- ---------- ---------- ---------- ---------- ---------- ---------- ---------- ---------- ---------- ---------- ---------- ---------- ---------- ---------- ---------- ----------
ELE               ---------- ---------- ---------- ---------- ---------- ---------- ---------- ---------- ---------- ---------- ---------- ---------- ---------- ---------- ---------- ---------- ---------- ---------- ---------- ----------
CH.1.3            ---------- ---------- ---------- ---------- ---------- ---------- ---------- ---------- ---------- ---------- ---------- ---------- ---------- ---------- ---------- ---------- ---------- ---------- ---------- ----------
CB                ---------- ---------- ---------- ---------- ---------- ---------- ---------- ---------- ---------- ---------- ---------- ---------- ---------- ---------- ---------- ---------- ---------- ---------- ---------- ----------
OMTai38           ---------- ---------- ---------- ---------- ---------- ---------- ---------- ---------- ---------- ---------- ---------- ---------- ---------- ---------- ---------- ---------- ---------- ---------- ---------- ----------
OMTai46           ---------- ---------- ---------- ---------- ---------- ---------- ---------- ---------- ---------- ---------- ---------- ---------- ---------- ---------- ---------- ---------- ---------- ---------- ---------- ----------
OMTai95           ---------- ---------- ---------- ---------- ---------- ---------- ---------- ---------- ---------- ---------- ---------- ---------- ---------- ---------- ---------- ---------- ---------- ---------- ---------- ----------
OMTai139          ---------- ---------- ---------- ---------- ---------- ---------- ---------- ---------- ---------- ---------- ---------- ---------- ---------- ---------- ---------- ---------- ---------- ---------- ---------- ----------
serow_AY149639    .T.....AT. .......... ......A..A .GC.TG..C. .C........ ......T... .......... .....A.... ...AAG.... ..-..C..C. .C........ ..A....... .......... .......... G.G....... .......... .......... ..A....A.. .......... ..........

                  605        615        625        635        645        655        665        675        685        695        705        715        725        735        745        755        765        775        785        795
CBL.6             CCCATTTCTT GTGGGGGTAG CTATTTAATG AACTTTATCA GACATCTGGT TCTTTCTTCA GGGCCATCTC ACCTAAAATC GCCCACTCTT TCCTC-TTAA ATAAGACATC TCGATGGACT AATGGCTAAT CAGCCCATGC TCACACATAA CTGTGATGTC ATACATTTGG TA-TTTTTAA TTTTTTGGGG GATGCTTGGA
CBL.7             .......... .......... .......... .......... .......... .......... .......... .......... .......... .....-.... .......... .......... .......... ....T..... .......... .......... .......... ..-....... .......... ..........
CBL.8             .......... .......... .......... .......... ......A... .......... .......... .......... .......... .....-.... .......... .......... .......... .......... .......... .......... .......... ..-....... .......... ..........
CBL.10            ......AA.C A...C...CA .......... .......... .......... .......... .......... .......... .......... .....C.... .......... .......... .......... .......... .......... .......... .......... ..-....... .......... ..........
CHL.4             .......... .......... .......... .......... .......... .......... .......... .......... .......... .....-.... ..G....... .......... .......... .......... .......... .......... .......... ..-C...... .......... ..........
CHL.5             ......AAC. .......... .......... .......... .......... .......... .......... .......... ......G... ...C.-.... .......... .......... ....A..... .......... .......... .......... .......... ..-....... .......... ..........
CHL.6             .......... .......... .......... .......... .......... .......... .......... .......... .......... .....-.... .......... .......... .......... .......... .......... .......... .......... ..-....... .......... ..........
CHL.7             .......... .......... .......... .......... .......... .......... .......... .......... .......... .....-.... .......... .......... .......... .......... .......... .......... .......... ..-....... .......... ..........
CHL.8             .......... .......... .......... .......... .......... .......... .......... .......... .......... .....-.... .......... .......... ....A..... .......... .......... .....C.... .......... ..T....... ....-..... ..........
EL.5              ......C... .......... .......... .......... .......... .......... .......T.. .T........ .......... .....-.... .......... .......... .G..A..... .......... .......... .....C.... .......... ..-....... .......... ..........
EL.6              ......C... .......... .......... .......... .......... .......... .......... .T........ .......... .....-.... .......... .......... .G..A..... .......... .......... .....C.... .......... ..-....... .......... ..........
EL.7              ......C... .......... .......... .......... .......... .......... .......... .T........ .......... .....-.... .......... .......... .G..A..... .......... .......... .....C.... .......... ..-....... .......... ..........
EL.8              ......C... .......... .......... .......... .......... .......... .......... .T........ .......... .....-.... .......... .......... .G..A..... .......... .......... .....C.... .......... ..-....... .......... ..........
EL.10             ......C... .......... .......... .......... .......... .......... .......... .T........ .......... .....-.... .......... .......... .G..A..... .......... .......... .....C.... .......... ..-....... .......... ..........
U47075            ---------- ---------- ---------- ---------- ---------- ---------- ---------- ---------- ---------- ---------- ---------- ---------- ---------- ---------- ---------- ---------- ---------- ---------- ---------- ----------
U47073            ---------- ---------- ---------- ---------- ---------- ---------- ---------- ---------- ---------- ---------- ---------- ---------- ---------- ---------- ---------- ---------- ---------- ---------- ---------- ----------
U47071            ---------- ---------- ---------- ---------- ---------- ---------- ---------- ---------- ---------- ---------- ---------- ---------- ---------- ---------- ---------- ---------- ---------- ---------- ---------- ----------
U47069            ---------- ---------- ---------- ---------- ---------- ---------- ---------- ---------- ---------- ---------- ---------- ---------- ---------- ---------- ---------- ---------- ---------- ---------- ---------- ----------
U47067            ---------- ---------- ---------- ---------- ---------- ---------- ---------- ---------- ---------- ---------- ---------- ---------- ---------- ---------- ---------- ---------- ---------- ---------- ---------- ----------
U47065            ---------- ---------- ---------- ---------- ---------- ---------- ---------- ---------- ---------- ---------- ---------- ---------- ---------- ---------- ---------- ---------- ---------- ---------- ---------- ----------
U47063            ---------- ---------- ---------- ---------- ---------- ---------- ---------- ---------- ---------- ---------- ---------- ---------- ---------- ---------- ---------- ---------- ---------- ---------- ---------- ----------
U47061            ---------- ---------- ---------- ---------- ---------- ---------- ---------- ---------- ---------- ---------- ---------- ---------- ---------- ---------- ---------- ---------- ---------- ---------- ---------- ----------
U47076            ---------- ---------- ---------- ---------- ---------- ---------- ---------- -----.GGCA TTA.TAATGC CATCTTCCTC TA.TA..-.. .......... .G..A..... ...-...... .......... .....C.... .......... ..--...... ....--.... ........-.
U47074            ---------- ---------- ---------- ---------- ---------- ---------- ---------. TT..C.GGCA TTA.TAATGC CATCTTCCTC TA.TA..-.. .......... .G..A..... ...-...... .......... .....C.... .......... ..--...... ....--.... ........-.
U47072            ---------- ---------- ---------- ---------- ---------- ---------- ---------. TT..C.GGCA TTA.TAATGC CATCTTCCTC TA.TA..-.. .......... .G..A..... ...-...... .......... .....C.... .......... ..--...... ....--.... ........-.
U47070            ---------- ---------- ---------- ---------- ---------- ---------- ---------- -----.GGCA TTA.TAATGC CATCTTCCTC TA.TA..-.. .......... .G..A..... ...-...... .......... .....C.... .......... ..--...... ....--.... ........-.
U47068            ---------- ---------- ---------- ---------- ---------- ---------- ---------. TT..C.GGCA TTA.TAATGC CATCTTCCTC TA.TA..-.. .......... .G..A..... ...-...... .......... .....C.... .......... ..--...... ....--.... ........-.
U47066            ---------- ---------- ---------- ---------- ---------- ---------- ---------. TT..C.GGCA TTA.TAATGC CATCTTCCTC TA.TA..-.. .......... .G..A..... ...-...... .......... .....C.... .......... ..--...... ....--.... ........-.
U47064            ---------- ---------- ---------- ---------- ---------- ---------- ---------. TT..C.GGCA TTA.TAATGC CATCTTCCTC TA.TA..-.. .......... .G..A..... ...-...... .......... .....C.... .......... ..--...... ....--.... ........-.
U47062            ---------- ---------- ---------- ---------- ---------- ---------- -------G.. TT..C.GGCA TTA.TAATGC CATCTTCCTC TA.TA..-.. .......... .G..A..... ...-...... .......... .....C.... .......... ..--...... ....--.... ........-.
CHLB              ---------- ---------- ---------- ---------- ---------- ---------- ---------- ---------- ---------- ---------- ---------- ---------- ---------- ---------- ---------- ---------- --------.. ..-....... .......... ..........
ELE               ---------- ---------- ---------- ---------- ---------- ---------- ---------- ---------- ---------- ---------- ---------- ---------- ---------- ---------- ---------- ---------- --------.. ..-....... .......... ..........
CH.1.3            ---------- ---------- ---------- ---------- ---------- ---------- ---------- ---------- ---------- ---------- ---------- ---------- ---------- ---------- ---------- ---------- --------.. ..-....... ...--G.... ..........
CB                ---------- ---------- ---------- ---------- ---------- ---------- ---------- ---------- ---------- ---------- ---------- ---------- ---------- ---------- ---------- ---------- --------.. ..-....... .....G.... ..........
OMTai38           ---------- ---------- ---------- ---------- ---------- ---------- ---------- ---------- ---------- ---------- ---------- ---------- ---------- ---------- ---------- ---------- --------.. ..-....... .......... ..........
OMTai46           ---------- ---------- ---------- ---------- ---------- ---------- ---------- ---------- ---------- ---------- ---------- ---------- ---------- ---------- ---------- ---------- --------.. ..-....... .......... ..........
OMTai95           ---------- ---------- ---------- ---------- ---------- ---------- ---------- ---------- ---------- ---------- ---------- ---------- ---------- ---------- ---------- ---------- --------.. ..-....... .......... ..........
OMTai139          ---------- ---------- ---------- ---------- ---------- ---------- ---------- ---------- ---------- ---------- ---------- ---------- ---------- ---------- ---------- ---------- --------.. ..-....... .......... ..........
serow_AY149639    ......AA.. .......... .......... ..T....... .......... .......... .......... .......... ......A... ...C.-.... .......... .......... ....A..... .......... .......... .....C.... .......... ..T....... ....--.... ..........
```

```
                 805        815        825        835        845        855        865        875        885        895        905        915        925        935        945        955        965        975        985        995
CBL.6            CTCAGCTATG GCC-GTCAAA GGCCCCGACT CGGAGCATAA ATTGTAGCTG GACTTAACTG CATCTTGAGC ACCCGCATAA TGGTAGGCGG GGACATCATA -ATTAATGGT CACAGGACAT ACTTGTTATA TATGCTCTT- -CGTACCCCC TCCTATCTTC CCTCCCCC-- TCCCCTTTTA -TATTTACAT CCATTCTTAA
CBL.7            .......... ...-...... .......... .......... .......... .......... .......... .......... .......... .......... -......... .......... .......... .........- -......... .......... ........-- .......... -......... ..........
CBL.8            .......... ...-...... .......... .......... .......... .......... .......... .......... .......... .......... -......... .......... .......... .........- -......... .......... ........-- .......... -......... ..........
CBL.10           .......... ...-...... .......... .......... .......... .......... .......... .......... .......... .......... -......... .......... .......... .........- -......... .......... ........-- .......... -......... ..........
CHL.4            .......... ...-...... .......... .......... .......... .......... .......... .......... .......... .......... -......... .......... .......... .........- -......... .......... ........-- .......... -......... ..........
CHL.5            .......... ...-...... .......... .......... .......... .......... .......... .......... .......... .......... -......... .......... .......... .........- -......... .......... ........-- .......... -......... ..........
CHL.6            .......... ...-...... .......... .......... .......... .......... .......... .......... .......... .......... -......... .......... .......... .........- -......... .......... ........-- .......... -......... ..........
CHL.7            .......... ...-...... .......... .......... .......... .......... .......... .......... .......... .......... -......... .......... .......... .........- -......... .......... ........-- .......... -......... ..........
CHL.8            .......... ...-...... .........C ........G. .......... ........C. .......... .......... ........AC .......... -......... .......... .ACCA...C. CTATGCAC.C C.......T. ..TAT...AT .T.T....CC .......... -......TC. .T........
EL.5             .......... ...-...... .........C .......... .......... .......... .......... .......... ........AA .......... -......... .......... ....A.C... .........- -......... .......... ........-- .......... -......... ..........
EL.6             .......... ...-...... .........C .......... .......... .......... .......... .......... ........AA .......... -......... .......... ....A.C... .........- -......... .......... ........-- .......... -......... ..........
EL.7             .......... ...-...... .........C .......... .......... .......... .......... .......... ........AA .......... -......... .......... ....A.C... .........- -......... .......... ........-- .......... -......... ..........
EL.8             .......... ...-...... .........C .......... .......... .......... .......... .......... ........AA .......... -......... .......... ....A.C... .........- -......... .......... ........-- .......... -......... ..........
EL.10            .......... ...-...... .........C .......... .......... .......... .......... .......... ........AA .......... -......... .......... ....A.C... .........- -......... .......... ........-- .......... -......... ..........
U47075           ---------- ---------- ---------- ---------- ---------- ---------- ---------- ---------- ---------- ---------- ---------- ---------- ---------- ---------- ---------- ---------- ---------- ---------- ---------- ----------
U47073           ---------- ---------- ---------- ---------- ---------- ---------- ---------- ---------- ---------- ---------- ---------- ---------- ---------- ---------- ---------- ---------- ---------- ---------- ---------- ----------
U47071           ---------- ---------- ---------- ---------- ---------- ---------- ---------- ---------- ---------- ---------- ---------- ---------- ---------- ---------- ---------- ---------- ---------- ---------- ---------- ----------
U47069           ---------- ---------- ---------- ---------- ---------- ---------- ---------- ---------- ---------- ---------- ---------- ---------- ---------- ---------- ---------- ---------- ---------- ---------- ---------- ----------
U47067           ---------- ---------- ---------- ---------- ---------- ---------- ---------- ---------- ---------- ---------- ---------- ---------- ---------- ---------- ---------- ---------- ---------- ---------- ---------- ----------
U47065           ---------- ---------- ---------- ---------- ---------- ---------- ---------- ---------- ---------- ---------- ---------- ---------- ---------- ---------- ---------- ---------- ---------- ---------- ---------- ----------
U47063           ---------- ---------- ---------- ---------- ---------- ---------- ---------- ---------- ---------- ---------- ---------- ---------- ---------- ---------- ---------- ---------- ---------- ---------- ---------- ----------
U47061           ---------- ---------- ---------- ---------- ---------- ---------- ---------- ---------- ---------- ---------- ---------- ---------- ---------- ---------- ---------- ---------- ---------- ---------- ---------- ----------
U47076           .......... ...A....C. .........C .......... .......... -......... .......... .......... ........AA .......... C......... .......... ....A.C... ..C......- -......... .......... ........-- .......... -......... ..........
U47074           .......... ...A...... .........C .......... .......... -......... .......... .......... ........AA .......... C......... .......... ....A.C... .........- -......... .......... ........-- .......... -......... ..........
U47072           .......... ...A...... .........C .......... .......... -......... .......... .......... ........AA .......... C......... .......... ....A.C... .........- -......... .......... ........-- .......... -......... ..........
U47070           .......... ...A...... .........C .......... .......... -......... .......... .......... ........AA .......... -......... .......... ....A.C... .........- -......... .......... ........-- .......... -......... ..........
U47068           .......... ...-...... .........C .......... .......... -......... .......... .T........ ........AA .......... -......... .......... ....A.C... .........- -......... .........- T.C.....-- .......... -......... ..........
U47066           .......... ...A...... .........C .......... .......... -......... .......... .......... ........AA .......... -......... .......... ....A.C... .........- -......... .......... ........-- .......... -......... ..........
U47064           .......... ...A...... .........C .......... .......... -......... .......... .......... ........AA .......... -......... .......... ....A.C... .........- -......... .......... ........-- .......... -......... ..........
U47062           .......... ...A...... .........C .......... .......... -......... .......... .......... ........AA .......... -......... .......... ....A.C... ..C......- -......... .......... ........-- .......... -......... ..........
CHLB             .......... ...-...... .......... .......... .......... .......... .......... .......... .......... .......... -......... .......... .......... .........- -......... .......... ........-- .......... -......... ........--
ELE              .......... ...-...... .........C .......... .......... .......... .......... .......... ........AA .......... -......... .......... ....A.C... .........- -......... .......... ........-- .......... -......... ........--
CH.1.3           .......... ...-A..T.. .....T...C .A.....GT. .......... .......... .......... .T.AC..... ........AC .AG....... -G.C...... .T.....T.. .AACA..... .CATA.A.CA CTCAT..... C..CCC.GGT TT.T....AC CTTAAA.A.C TACCACCAT. TTT-------
CB               .......... ...-...... .......... .......... .......... .......... .......... .......... ........AA .......... -......... .......... ....A..... .........- -........T .......... ........-- .......... -......... .T......--
OMTai38          .......... ...-...... .......... .......... .......... .......... ..C....... .......... ........AA ..G....... -......... .G........ ...CA..... .........- -......... .......... ........-- .......... -......... .T......--
OMTai46          .......... ...-...... .........C .......... .......... .......... ..C....... .......... ........AA .......... -......... .G....G... ...CA..... .........- -......... .......... ........-- .......... -......... .T......--
OMTai95          .......... ...-...... .........C .......... .......... .......... .......... .......... .........A .......... -......... .G........ ...CA..... .........- -......... .......... ........-- .......... -......... .T......--
OMTai139         .......... ...-...... .........C .......... .......... .......... ..CT...... .......... ........AA .......... -......... .......... ...CA..... .........- -......... .......... ........-- .......... -......... .T......--
serow_AY149639   .......... ...-...... .........C .......... .......... .......... .......... .T..C..... ........AT ..G....... -......... .......... ...-A..... ACATGCA.CC C......T.T C.GCT.TA.T ..C.....-- .T...C.... A....C..C. .T........

                 1005       1015       1025       1035       1045       1055       1065       1075       1085       1095       1105       1115       1125       1135
CBL.6            CACACTCATC CCTAGATATA AACCTAAATT TATCCTCTCT TCAATACTCA AATCTGCACT CCAACCAAGA TAGACATATA GGCACCTGGG TCTTCCCCAT AAAAC--AGT TAATGTAGCT TAAAATTAAA GCAAGG
CBL.7            .......... .......... .......... .......... .......... .......... .......... .......... .......... .......... .....--... ...C...... .......... ......
CBL.8            .......... .......... .......... C......... .......... .......... .......... .......... .......... .......... .....--... .......... .......... ......
CBL.10           .......... .......... .......... .......... .......... .......... .......... .......... .......... .......... .....--... .......... .......... ......
CHL.4            .......... .......... .......... .......... .......... .......... .......... .......... .......... .......... .....--... .......... .......... ......
CHL.5            .......... .......... .......... .......... .......... .......... .......... .........G .......... .......... .....--... .......... .......... ......
CHL.6            .......... .......... .......... .......... .......... .......... .......... .......... .......... .......... .....--... .......... .......... ......
CHL.7            .......... .......... .......... .......... .......... .......... .......... .......... .......... .......... .....--... .......... .......... ......
CHL.8            .......... .......... .......... ...T.C...C .......... ....C..... .........G .......... .......... ......T... .....AT... .......... .......... ......
EL.5             .......... .......... .......... .......... .......... .......... .......... .......... .......... .......... .....--... .....C.... .......... ......
EL.6             .......... .......... .......... .......... .......... .......... .......... .......... .......... .......... .....--... .......... .......... ......
EL.7             .......... .......... .......... .......... .......... .......... .......... .......... .......... .......... .....--... .......... .......... ......
EL.8             .......... .......... .......... .......... .......... .......... .......... .......... .......... .......... .....--... .......... .......... ......
EL.10            .......... .......... .......... .......... .......... .......... .......... .......... .......... .......... .....--... .......... .......... ......
U47075           ---------- ---------- ---------- ---------- ---------- ---------- ---------- ---------- ---------- ---------- ---------- ---------- ---------- ------
U47073           ---------- ---------- ---------- ---------- ---------- ---------- ---------- ---------- ---------- ---------- ---------- ---------- ---------- ------
U47071           ---------- ---------- ---------- ---------- ---------- ---------- ---------- ---------- ---------- ---------- ---------- ---------- ---------- ------
U47069           ---------- ---------- ---------- ---------- ---------- ---------- ---------- ---------- ---------- ---------- ---------- ---------- ---------- ------
U47067           ---------- ---------- ---------- ---------- ---------- ---------- ---------- ---------- ---------- ---------- ---------- ---------- ---------- ------
U47065           ---------- ---------- ---------- ---------- ---------- ---------- ---------- ---------- ---------- ---------- ---------- ---------- ---------- ------
U47063           ---------- ---------- ---------- ---------- ---------- ---------- ---------- ---------- ---------- ---------- ---------- ---------- ---------- ------
U47061           ---------- ---------- ---------- ---------- ---------- ---------- ---------- ---------- ---------- ---------- ---------- ---------- ---------- ------
U47076           .......... .......... .......... .......... .......... .......... .......... .......... .......... .......... .....--... .......... .......... ......
U47074           .......... .......... .......... .......... .......... .......... .......... .......... .......... .......... .....--... .......... .......... ......
U47072           .......... .......... .......... .......... .......... .......... .......... .......... .......... .......... .....--... .......... .......... ......
U47070           .......... .......... .......... .......... .......... .......... .......... .......... .......... .......... .....--... .......... .......... ......
U47068           .......... .......... .......... .......... .......... .......... .......... .......... .......... .......... .....--... .......... .......... ......
U47066           .......... .......... .......... .......... .......... .......... .......... .......... .......... .......... .....--... .......... .......... ......
U47064           .......... .......... .......... .......... .......... .......... .......... .......... .......... .......... .....--... .......... .......... ......
U47062           .......... .......... .......... .......... .......... .......... .......... .......... .......... .......... .....--... .......... .......... ......
CHLB             ---------- ---------- ---------- ---------- ---------- ---------- ---------- ---------- ---------- ---------- ---------- ---------- ---------- ------
ELE              ---------- ---------- ---------- ---------- ---------- ---------- ---------- ---------- ---------- ---------- ---------- ---------- ---------- ------
CH.1.3           ---------- ---------- ---------- ---------- ---------- ---------- ---------- ---------- ---------- ---------- ---------- ---------- ---------- ------
CB               ---------- ---------- ---------- ---------- ---------- ---------- ---------- ---------- ---------- ---------- ---------- ---------- ---------- ------
OMTai38          ---------- ---------- ---------- ---------- ---------- ---------- ---------- ---------- ---------- ---------- ---------- ---------- ---------- ------
OMTai46          ---------- ---------- ---------- ---------- ---------- ---------- ---------- ---------- ---------- ---------- ---------- ---------- ---------- ------
OMTai95          ---------- ---------- ---------- ---------- ---------- ---------- ---------- ---------- ---------- ---------- ---------- ---------- ---------- ------
OMTai139         ---------- ---------- ---------- ---------- ---------- ---------- ---------- ---------- ---------- ---------- ---------- ---------- ---------- ------
serow_AY149639   .......G.. .......... ..T....... ...T.CTC.C C......... ...TG..... .T......AG C.A....... ...G...... ...C...... .G.C.AT... .......... .......... ......
```
