# Supplementary material for: Detection of mitochondrial insertions in the nucleus (NuMts) of Pleistocene and modern muskoxen
Source: BMC Evol Biol. 2007 Apr 27;7:67. doi: 10.1186/1471-2148-7-67 (PMC1876215; doi:10.1186/1471-2148-7-67)
Supplement: Additional file 4 — Explanation of haplotypes. Collapsed sequences into haplotypes treating gaps as 5th state. The frequency is calculated within the same individual muskox and clone type (e.g. hair or blood). [file 1471-2148-7-67-S4.doc]

**Additional Table 2**

| **Haplotype name** | **Collapsed sequences** | **Frequency** |
| --- | --- | --- |
| MMO235 | MMO2, MMO3, MMO5 | 0.375 |
| MMO67 | MMO6, MMO7 | 0.25 |
| OMtai1439 | OMTai14, OMTai39 | 0.4 |
| CLHB | CHL.1- 3, 5-7, CBL.3-10 | 0.75, 0.8 |
| CHB | CH.1.8, CB.1.10 | 0.1, 0.1 |
| CB | CB.1, 2, 7, 8 | 0.4 |
| CB.1.69 | CB.1, 6, 9 | 0.3 |
| ELE | EL.1-9, E.2, 3, 6, 8-10 | 1.0, 0.6 |
